# Daytime sleepiness and specific food cravings: The moderating role of insulin sensitivity

**DOI:** 10.1371/journal.pone.0343407

**Published:** 2026-03-09

**Authors:** Emma K. Romaker, Mason J. Krueger, Mollie S. Goldfinger, Alex Gonzalez, Daniel E. Forster, William K. Wohlgemuth, Roger C. McIntosh, Barry E. Hurwitz

**Affiliations:** 1 Department of Psychology, University of Miami, Coral Gables, Florida, United States of America; 2 Behavioral Medicine Research Center, Miller School of Medicine, University of Miami, Miami, Florida, United States of America; 3 Psychology and Neurology Service, Bruce W. Carter Medical Center, Miami VA Healthcare System, Miami, Florida, United States of America; 4 Division of Endocrinology, Diabetes and Metabolism, Miller School of Medicine, University of Miami, Miami, Florida, United States of America; University of Leipzig Faculty of Life Sciences: Universitat Leipzig Fakultat fur Lebenswissenschaften, GERMANY

## Abstract

**Purpose:**

Daytime sleepiness is posited to stimulate hunger and food intake of specific macronutrients such that obesity and type 2 diabetes mellitus risk may be elevated. To assess this hypothesis, this study of insulin sensitive and insulin resistant non‑diabetic individuals utilized standardized meal administration conditions to examine: 1) the extent to which self-reported sleepiness was associated with specific food cravings over the course of a day; and 2) whether insulin sensitivity interactively influenced this relationship.

**Methods:**

Non-diabetic men and women (N = 143) participated in one session, where a euglycemic-hyperinsulinemia clamp was used to provide an insulin sensitivity index, and in a subsequent 14‑hour session, where four standardized mixed-meals and one pre-bedtime meal were provided. Concurrent pre-meal measures of sleepiness and cravings for sweet, salty, and starchy foods, and fruit, meat, and dairy foods were obtained. Hierarchical linear modeling analyses examined the within- and between-person association of sleepiness with food cravings across meals as a function of insulin sensitivity, controlling for age, sex and caloric intake administration.

**Results:**

Craving ratings were highest for fruits, followed by dairy and starchy foods, meat, and then salty and sweet foods (p < .001). Analyses showed that insulin sensitivity moderated the positive association of daytime sleepiness with all food cravings, except for salty foods (p = .011 to .036), independent of covariates. This moderation effect displayed the strongest magnitude at below-average and average insulin sensitivity levels (p < .001).

**Conclusions:**

Study results extend previous findings to show that daytime sleepiness is positively associated with cravings for a range of food types. The fact that these associations were increased in persons with more diminished insulin sensitivity is novel and supports further examination of underlying mechanisms linking daytime sleepiness and food cravings with food consumption and metabolic dysregulation early in diabetes pathophysiology.

## Introduction

Many adults in the United States (US) lack consistent, sufficient sleep, commonly resulting in daytime sleepiness, diminished cognitive function, interference with daily activities, and increased risk for obesity and type 2 diabetes [1–5]. A related line of research suggests that poorer sleep function stimulates food seeking and intake, and alters specific food intake, which in turn supports metabolic dysregulation and fat deposition [6]. For example, sleep restriction to 4 hours/night compared with sleeping 10 hours/night resulted in increased food consumption [7]. In another study, when sleep was restricted to 5.5 hours/night, snacking and carbohydrate intake was greater than when sleep was permitted for 8 hours/night [8]. Others have shown that sleep restriction (6.5 hours in bed) compared with sleep extension (10 hours in bed) resulted in greater consumption of sweets and desserts [9]. In contrast, in persons displaying habitually shorter sleep duration compared with those sleeping longer, eating snacks in place of meals correlated with consuming more energy‑dense foods (i.e., fat and sweets) and less fruits and vegetables [10]. Similarly, in individuals habitually sleeping less than 8 hours/night, higher fat consumption and snacking have also been observed [11]. Thus, studies of non-diabetic persons have shown that sleep restricting manipulations and habitual short sleep patterns are linked with altered food preferences and intake of specific food types.

A food craving, which may also be referred to as a selective food hunger or preference, is a term used to describe a desire of significant intensity to consume a specific food [12]. Whether food cravings vary as a function of daytime wakefulness and sleepiness, arising from accumulated sleep load, circadian influences, and related factors, remains unclear [13]. Sleep‑wake dysregulation has, however, been linked to metabolic alterations relevant to energy balance [6]. Notably, insulin resistance can emerge prior to the development of prediabetes and well in advance of a formal diagnosis of type 2 diabetes [14]. The progressive accumulation of adipose tissue in visceral and hepatic compartments is associated with worsening insulin resistance, indicating a close quantitative relationship between peripheral and hepatic insulin sensitivity [14]. Despite these established metabolic links, prior research has not examined whether insulin sensitivity modifies the association between sleepiness and food craving, an interaction that may be mechanistically relevant to obesity, prediabetes, and type 2 diabetes pathophysiology [see S1 File].

To address these scientific gaps, the current study of non-diabetic individuals, evaluated whether insulin sensitivity, as an integrated metabolic status marker that includes adiposity-related variance, influenced the association of daytime sleepiness with specific food cravings (i.e., hunger for sweet, salty, and starchy foods, fruit, meat, and dairy) over a 14-hour period, in controlled conditions, following an overnight in-laboratory stay. It was hypothesized that higher daytime sleepiness levels would correspond with higher cravings (specifically for sweet, starchy, and calorie dense foods), and that this relationship would be strengthened in participants with more diminished insulin sensitivity.

## Materials and methods

### Participants

The cohort of 143 men and women were recruited from South Florida [15]. Study eligibility criteria included: 18–55 years of age, no nicotine use in the year prior to study entry, no history or current dependency on substances or alcohol, negative urine toxicology screen, normative thyroid stimulating hormone level, no prior diagnosis of cardiovascular, cholesterol, psychiatric, endocrine or sleep condition, and no prescribed medications for these or other conditions. Women were premenopausal with regular menstrual cycling (26–35 days).

### Ethical approval

The study recruitment took place between April 1, 2007 and April 30, 2010. The study protocol was approved by the institutional review board of the University of Miami. Written informed consent was obtained from participants prior to inclusion.

### Study design and procedures

This study is a secondary analysis of a previous study, which assessed cardiometabolic function in nondiabetic persons with and without insulin resistance [15, for a detailed summary of procedures]. In sum, in study-eligible individuals, demographic, anthropometric, personal medical history, and standard cardiometabolic risk factor information was collected. In addition, a euglycemic-hyperinsulinemia clamp was performed to determine insulin sensitivity. During a 3‑day, overnight laboratory stay, participants were provided four meals per day, with a meal provided every 3.5 hours over 14‑hour periods. On the first day, body fat deposition and cardiometabolic measurements were obtained, and an overnight fast followed [15]. On the second and third days, mixed-meals were given at 8:00 am (Meal 1), 11:30 am (Meal 2), 3:00 pm (Meal 3), and 6:30 pm (Meal 4), and a snack at 10:00 pm (Meal 5). The mixed-meal macronutrient content was adjusted for sex, body surface area, and the sedentary energy expenditure expected during the laboratory stay. The intent was to approximate the average U.S. national daily total caloric intake and macronutrient composition (i.e., 50% carbohydrate, 35% fat and 15% protein) [16]. The percent composition of fat and protein calories was identical for each meal. However, the caloric intake per day was manipulated so that 50% greater calories were consumed on one day than the other day. This difference was achieved by manipulating the meal carbohydrate content so that on one day participants received a standard level of carbohydrate intake per meal (~300 kcal/meal), and on the other day participants received a double portion of carbohydrate content per meal (~600 kcal/meal).

### Daytime sleepiness and food cravings

Both sleepiness and specific food cravings were measured by self-report using a visual analog scale from 0 mm (“not at all”) to 100 mm (“very much”). Ratings were collected immediately prior to each meal/snack administration. When rating sleepiness, participants were asked how sleepy they currently felt. When rating food cravings, participants were asked to rate their current appetite for specific food types. These ratings were performed serially, in fixed order, for six different food types: sweets (e.g., cake, cookies, candy), salty (e.g., chips, salted nuts, pickles), starchy (e.g., bread, pasta, cereal), meat (e.g., hamburger, steak, chicken), fruit (e.g., apples, oranges, pineapple), and dairy (e.g., milk, cheese, yogurt). The fixed sequence of the survey questions was undertaken to preserve comparability with prior research, optimize participant comprehension, and reduce participant burden.

### Insulin sensitivity

The euglycemic-hyperinsulinemia clamp involved a priming insulin infusion, followed by a constant insulin infusion at 40 μU/m^2^/min for 150 minutes using a calibrated IMed Gemini PC-2TX infusion pump (Alaris Medical Systems, San Diego, CA) [15]. Glucose infusion was undertaken 4‑minutes after insulin infusion began, and blood glucose was clamped thereafter within 5% of fasting levels. During the steady state phase, the glucose infusion rate is equal to the rate of total‑body glucose uptake. Insulin sensitivity was indexed by the whole-body glucose disposal rate (mg/kg•min of fat-free mass). Insulin resistance status was defined as a value at or lower than 4.5 mg/kg•min [17].

### Covariates

Age, sex, and race/ethnicity were obtained through self-report. Race/ethnicity was coded as Hispanic (0), non-Hispanic White (1), Black (2), and Other (3). Meal administration sequence was determined by the random assignment to the 2-day carbohydrate-loading manipulation (i.e., standard-double vs. double-standard). For the main effect models, insulin sensitivity was included as a covariate.

### Statistical analysis

Data screening involved visual examination (i.e., boxplot, QQ plot, histogram of residuals and random effects, and scatterplots) for normal distribution of residuals at the within-person level, the normal distribution of random effects at the between-person level, and evidence of heteroscedasticity at the within-person level (due to sleepiness) and at the between-person level (due to insulin sensitivity). Descriptive statistics and analyses of covariance (ANCOVA) were conducted using SPSS (v28.0) to examine demographic and metabolic characteristic differences between participants classified as insulin sensitive (IS) and insulin resistant (IR). Because hunger would be expected to be suppressed on the high caloric intake day, wherein caloric intake was 50% higher than the standard day, the current analyses were restricted to the standard caloric-intake day. Thus, study results may more closely reflect typical daily life experience unobscured by high caloric intake. ANCOVA analyses examined differences, as a function of IS and IR group status, among the 5-repeated food cravings measures per meal, as well as using AUC measures of the food cravings across the standard caloric-intake day. The trapezoidal method was used to calculate AUC0 and AUCi of the food cravings per time of meal, wherein the AUC0 was quantified by computing the total area under the curve from 0 across meals, and the AUCi was derived by computing the net incremental area under the curve across meals 2–5 relative to the pre‑meal 1 rating. Thus, the AUC0 provided an index of total craving magnitude, whereas the AUCi provided an index of craving change across the day relative to the meal 1 post-overnight fasting craving level.

Hierarchical linear modeling (HLM) was used to assess the association of sleepiness with food cravings across meals, while controlling for covariates. HLM was used because it can simultaneously disentangle relationships within- and between-hierarchical levels of nested data [18,19]. These analyses were performed using R (v4.1.2), and employed the lmer function, of the “lme4” package [18]. Notably, separate HLM analyses for each food craving were conducted [see S1 File, hypothesized model], which included the main effect of sleepiness, the within- and between-person effects of sleepiness, the interaction of insulin sensitivity for both within- and between-person sleepiness rating levels, and covariates. Within-person sleepiness ratings for each participant were calculated by subtracting the sleepiness rating at each meal from the grand mean of the participant across meals; thus, this effect tested differences at the individual level in the fluctuation of the associations across meals. In addition, between-person sleepiness ratings for each participant were calculated by subtracting the sleepiness rating at each meal from the total sample grand mean across meals; thus, this effect tested differences among individuals in the fluctuation of the associations relative to the sample mean [19]. Given that the craving rating measures were repeatedly collected prior to each meal at equal intervals, 8:00 am (0), 11:30 am (1), 3:00 pm (2), 6:30 pm (3), and 10:00 pm (4), the Meal‑time variable was modeled categorically. Due to the observed quadratic trend in specific food cravings over time, a squared term for the Meal-time variable was added to the models. Models were estimated using full information maximum likelihood. Results are reported as unstandardized estimates (β) ±SE, and significance was set to p < 0.05. Significant interactions were followed up with simple slope analyses at ±1 SD of insulin sensitivity.

## Results

### Participant characteristics

Of 833 persons screened, 690 persons were ineligible, declined to participate or were not available for follow-up. Thus, the final cohort comprised 143 participants. Complete data were obtained on all study parameters analyzed. Preliminary analyses of study measures indicated no evidence of heteroscedasticity or normality deviations. Thus, variable transformations were not warranted. Of the total sample, 45% were classified as IS, with the remainder classified as IR. Participant characteristics for IS and IR groups are summarized in Table 1. As can be seen in this table, no significant group differences were observed in age, sex, or meal administration sequence. Furthermore, the groups did not significantly differ on other demographic variables, including education, income, and race/ethnicity composition, when controlling for covariates. The cohort was largely comprised of minority individuals; of the cohort, about 74% were Hispanic persons and 16% Black persons. The IR group, compared with IS counterparts, displayed significantly higher BMI, wherein about 97% of the IR group were overweight or obese, whereas about 60% of the IS group were overweight or obese. In addition, the IR group demonstrated about 3-fold lower insulin sensitivity, with significantly higher fasting insulin and glucose levels than the IS group, independent of covariates.

**Table 1:** Descriptive statistics, mean or % (SE), for demographic and metabolic characteristics comparing insulin sensitive and insulin resistant groups.

	Overall (N = 143)	Insulin Sensitive (n = 64)	Insulin Resistant (n = 79)	Difference (p)
Age (years)	38.7 (0.7)	37.2 (1.0)	39.9 (1.0)	.06
Sex (n, % male)	93 (65.0)	41 (64.1)	52 (65.8)	.83
Sequence (n, % standard to high)	72 (50.4)	30 (46.9)	42 (53.2)	.46
Race/Ethnicity (n, %)				.41
Hispanic	106 (74.1)	45 (70.3)	61 (77.2)	
Black	23 (16.1)	9 (14.1)	14 (17.7)	
Non-Hispanic White	9 (6.3)	7 (10.9)	2 (2.5)	
Other	5 (3.5)	3 (4.7)	2 (2.5)	
Education (years)^**a**^	13.3 (0.2)	13.1 (0.3)	13.4 (0.3)	.50
Body Mass Index (kg/m^2^)^**a**^	29.0 (0.34)	26.3 (0.5)	31.7 (0.4)	**<.001**
Body Mass Index Classification				**<.001**
Normal weight (n, %)	28 (19.6)	26 (40.6)	2 (2.5)	
Overweight (n, %)	56 (39.2)	28 (43.8)	28 (35.4)	
Obese (n, %)	59 (41.3)	10 (15.6)	49 (62.0)	
Fasting Insulin (µU/L)^**a**^	12.8 (0.6)	9.7 (0.8)	16.0 (0.8)	**<.001**
Fasting Glucose (mg/dL)^**a**^	89.1 (0.8)	87.1 (1.2)	91.1 (1.1)	**<.02**
Insulin Sensitivity (mg/kg•min)^**a**^	5.2 (0.2)	7.7 (0.3)	2.8 (0.2)	**<.001**

^**a**^ analysis controlled for age, sex race/ethnicity and sequence.

**Fig 1:**
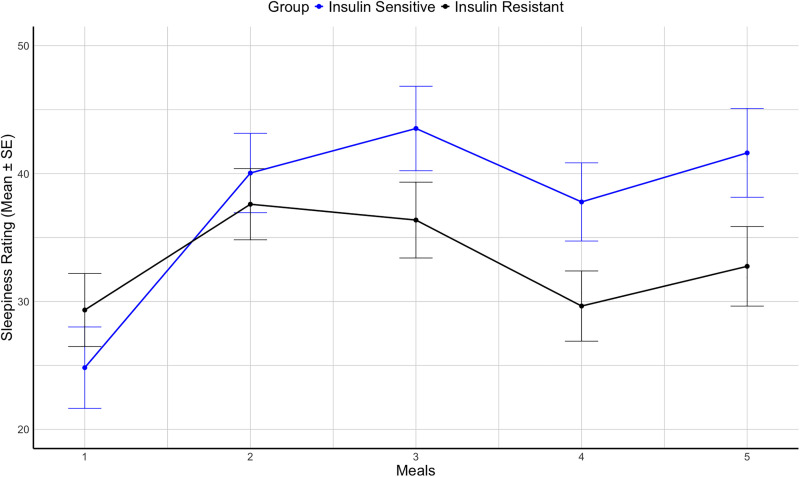
Sleepiness ratings across meals by group (insulin sensitive vs. insulin resistant). Reported as mean ±SE, adjusted for age, sex, race/ethnicity, and meal administration sequence. Meals were administered at: meal 1 (8:00 am), meal 2 (11:30 am), meal 3 (3:00 pm), meal 4 (6:30 pm), meal 5 (10:00 pm).

An ANCOVA analysis of sleepiness ratings across the meals was conducted comparing IS and IR groups, controlling for covariates. As shown in Fig 1, a significant interaction between groups and meals was observed (*F*(1,135 = 6.4, *p* < .02). Follow‑up analyses showed that there were no significant differences between groups from meal 1 to meal 3, but the IS group, relative to the IR group, reported significantly greater sleepiness on meals 4 and 5 (*F*(1,135 = 5.1, *p* < .03); for these meals, respective mean (±SE) sleepiness ratings for the IS and IR groups was 39.7 ± 2.8 and 31.2 ± 2.5.

The ANCOVA assessing all of the food craving ratings across meals comparing IS and IR groups, while controlling for covariates, revealed a significant interaction between cravings and meals (*F*(1,135 = 4.3, *p* < .04), but no main effect or interaction involving IS vs. IR grouping. As seen in Table 2, each of the food cravings displayed quadratic trends across meals, wherein ratings increased from meal 1 and maximized at meals 2 and 3, and, thereafter, progressively declined from meal 4 to meal 5. Post hoc analysis showed that this interaction primarily resulted from differences among craving ratings at meal 1 (*F*(1,135 = 17.7, *p* < .001). Specifically, at meal 1, craving ratings were highest for fruits, followed by dairy foods, starchy foods, meat, and then salty and sweet foods. Notably, the meal 1 craving for fruits were more than 2.5-fold greater than for sweet foods. Thus, while differences in cravings were observed following an overnight fasting period, all cravings displayed an increase at mid-day and then recovered to or close to the pre-meal 1 levels by the end of the day.

**Table 2:** Food craving ratings^a^ across meal administrations.

Food Type	Mean	M1	M2	M3	M4	M5
Sweets (mm)	29.4 (1.6)	16.9 (1.7)	32.5 (2.1)	37.5 (2.4)	35.8 (2.3)	24.3 (2.2)
Salty (mm)	33.1 (1.7)	19.8 (1.8)	41.7 (2.4)	40.2 (2.4)	39.9 (2.3)	24.0 (2.1)
Starchy (mm)	36.9 (1.7)	28.0 (2.2)	45.7 (2.3)	43.0 (2.3)	41.5 (2.3)	26.3 (2.2)
Meat (mm)	40.2 (1.8)	23.0 (1.9)	57.1 (2.4)	49.7 (2.5)	46.6 (2.4)	24.6 (2.2)
Fruit (mm)	49.0 (2.0)	42.6 (2.6)	56.1 (2.3)	54.7 (2.4)	51.2 (2.5)	40.5 (2.5)
Dairy (mm)	40.7 (2.0)	37.4 (2.6)	45.8 (2.3)	43.4 (2.4)	41.5 (2.4)	35.3 (2.5)

Abbreviations: M1 = meal 1, M2 = meal 2, M3 = meal 3, M4 = meal 4, M5 = meal 5.

^**a**^ Values reported as mean (±SE), adjusted for covariates (age, sex, race/ethnicity, and meal administration sequence); craving ratings are interpretable as 0 mm (“not at all”) to 100 mm (“very much”).

Of note, compared with the IR group, across all craving ratings, the IS group displayed significantly greater increase in AUCi relative to meal 1 (*F*(1,135 = 5.7, *p* < .02; AUCi mean ±SE: IS was 23,407.4 ± 2062.8; IR was 16,694.5 ± 1851.6). Otherwise, no significant difference among food craving ratings across meals was observed in overall magnitude of cravings indexed by AUC0 or the change in craving ratings, indexed by AUCi.

### Insulin sensitivity and the sleepiness-food craving association across meals

The HLM analyses revealed significant within-person main effects of sleepiness: sweet foods (β*(SE)*=0.17(0.04), *p* < .001), salty foods (β*(SE)*=0.14(0.04), *p* < .001), starchy foods (β*(SE)*=0.15(0.04), *p* < .001), meat (β*(SE)*=0.11(0.04), *p* < .01), fruit (β*(SE)*=0.17(0.04), *p* < .001), and dairy foods (β*(SE)*=0.15(0.04), *p* < .001). Thus, across the meals, with greater sleepiness relative to an individual’s average sleepiness, food craving ratings were higher. However, the within‑person effect of sleepiness ratings was not significantly moderated by insulin sensitivity. The random-slopes specification for Meal-time was not generally supported across outcomes, and substantive conclusions were not changed when random-slopes were used in modeling analyses. Thus, the fixed-slope specification for Meal-time was used for all analyses for parsimony and consistency. Final modeling results for each food craving are presented in Tables 3–8.

**Table 3:** Model assessing the insulin sensitivity moderation of the association of sleepiness with cravings for sweet food.^a^

Parameter	Main Effect	Within Person	Between Person	Interaction
Intercept	**1.52 (.31)** ^***^	**2.28 (.29)** ^***^	**2.28 (.29)** ^***^	**2.24 (.28)** ^***^
Age	.01 (.02)	.01 (.02)	.01 (.02)	.01 (.02)
Sex	−.43 (.32)	−.47 (.31)	−.47 (.31)	−.40 (.31)
Sequence	**−.60 (.30)** ^*^	−.51 (.29)	−.51 (.29)	−.48 (.28)
Race/Ethnicity	.001 (.17)	−.07 (.17)	−.07 (.17)	−.06 (.17)
IS	.02 (.06)	.01 (.05)	.01 (.05)	.01 (.05)
Meal-time	**1.72 (.17)** ^***^	**1.76 (.17)** ^***^	**1.76 (.17)** ^***^	**1.76 (.17)** ^***^
Sleepiness	.22 (.03)^***^	–	–	–
Meal-time^2^	**−.39 (.04)** ^***^	**−.40 (.04)** ^***^	**−.40 (.04)** ^***^	**−.40 (.04)** ^***^
W-P	–	**.17 (.04)** ^***^	–	**.17 (.04)** ^***^
B-P		–	**.47 (.08)** ^***^	**.45 (.08)** ^***^
W-P *IS	–	–	–	−.01 (.01)
B-P*IS	–	–	–	**−.06 (.03)** ^*^
**Variance Components**				
Intercept (*u*_0i_)	2.44	2.24	2.24	2.16
Residual (*e*_ti_)	3.27	3.26	3.26	3.26

Abbreviations: IS = insulin sensitivity, W-P = within-person sleepiness, B-P = between-person sleepiness.

^a^ Values reported as unstandardized estimates (β) ±SE; the β values reflect rescaled scores. Intercept is interpretable at grand mean centered age (x̄ = 39 years), dummy coded caloric loading (standard coded 0 as reference), dummy coded race/ethnicity (Hispanic coded 0 as reference), grand mean centered body mass index [BMI] (x̄ = 29.27 kg/m^2^), dummy coded Meal-time variable (meal 1 as reference), dummy coded sex (male coded 0 as reference), insulin sensitivity of 5.15 mg/kg•min, and sleepiness, (x̄ = 35.12 mm).

* *p* < .05, ** *p* < .01, *** *p* < .001.

**Table 4:** Model assessing the insulin sensitivity moderation of the association of sleepiness with cravings for salty food.^a^

Parameter	Main Effect	Within Person	Between Person	Interaction
Intercept	**2.16 (.33)** ^***^	**2.80 (.30)** ^***^	**2.80 (.30)** ^***^	**2.79 (.30)** ^***^
Age	−.02 (.02)	−.02 (.02)	−.02 (.02)	−.02 (.02)
Sex	**−.86 (.35)** ^*^	**−.91 (.33)** ^**^	**−.91 (.33)** ^**^	**−.86 (.33)** ^*^
Sequence	−.53 (.32)	−.43 (.30)	−.43 (.30)	−.40 (.30)
Race/Ethnicity	−.05 (.19)	−.14 (.18)	−.14 (.18)	−.13 (.18)
IS	.06 (.05)	.05 (.05)	.05 (.05)	.05 (.05)
Meal-time	**2.01 (.17)** ^***^	**2.05 (.17)** ^***^	**2.05 (.17)** ^***^	**2.04 (.17)** ^***^
Sleepiness	**.19 (.03)** ^***^	–	–	–
Meal-time^2^	**−.49 (.04)** ^***^	**−.50 (.04)** ^***^	**−.50 (.04)** ^***^	**−.50 (.04)** ^***^
W-P	–	**.14 (.04)** ^***^	–	**.13 (.04)** ^***^
B-P	–	–	**.48 (.08)** ^***^	**.46 (.08)** ^***^
W-P *IS	–	–	–	.02 (.01)
B-P*IS	–	–	–	−.05 (.03)
**Variance Components**				
Intercept (*u*_0i_)	2.86	2.59	2.59	2.52
Residual (*e*_ti_)	3.33	3.32	3.32	3.30

Abbreviations: IS = insulin sensitivity, W-P = within-person sleepiness, B-P = between-person sleepiness.

^a^ Values reported as unstandardized estimates (β) ±SE; the β values reflect rescaled scores. Intercept is interpretable at grand mean centered age (x̄ = 39 years), dummy coded caloric loading (standard coded 0 as reference), dummy coded race/ethnicity (Hispanic coded 0 as reference), grand mean centered body mass index [BMI] (x̄ = 29.27 kg/m^2^), dummy coded Meal-time variable (meal 1 as reference), dummy coded sex (male coded 0 as reference), insulin sensitivity of 5.15 mg/kg•min, and sleepiness, (x̄ = 35.12 mm).

* *p* < .05, ** *p* < .01, *** *p* < .001.

**Table 5:** Model assessing the insulin sensitivity moderation of the association of sleepiness with cravings for starchy food.^a^

Parameter	Main Effect	Within Person	Between Person	Interaction
Intercept	**3.26 (.33)** ^***^	3.95 (.30)***	**3.95 (.30)** ^***^	**3.93 (.30)** ^***^
Age	.01 (.02)	.01 (.02)	.01 (.02)	.01 (.02)
Sex	**−1.30 (.34)** ^***^	**−1.34 (.33)** ^***^	**−1.34 (.33)** ^***^	**−1.27 (.32)** ^***^
Sequence	**−.70 (.31)** ^*^	**−.60 (.30)** ^*^	**−.60 (.30)** ^*^	−.57 (.30)
Race/Ethnicity	−.26 (.18)	−.34 (.18)	−.34 (.18)	−.33 (.17)
IS	.05 (.05)	.04 (.05)	.04 (.05)	.04 (.05)
Meal-time	**1.58 (.18)** ^***^	**1.63 (.18)** ^***^	**1.63 (.18)** ^***^	**1.62 (.18)** ^***^
Sleepiness	**.21 (.04)** ^***^	–	–	–
Meal-time^2^	**−.42 (.04)** ^***^	**−.43 (.04)** ^***^	**−.43 (.04)** ^***^	**−.43 (.04)** ^***^
W-P	–	**.15 (.04)** ^***^	–	.14 (.04)^***^
B-P	–	–	**.50 (.08)** ^***^	**.48 (.08)** ^***^
W-P *IS	–	–	–	.01 (.01)
B-P*IS	–	–	–	**−.07 (.03)** ^*^
**Variance Components**				
Intercept (*u*_0i_)	2.72	2.44	2.44	2.35
Residual (*e*_ti_)	3.66	3.65	3.65	3.64

Abbreviations: IS = insulin sensitivity, W-P = within-person sleepiness, B-P = between-person sleepiness.

^a^ Values reported as unstandardized estimates (β) ±SE; the β values reflect rescaled scores. Intercept is interpretable at grand mean centered age (x̄ = 39 years), dummy coded caloric loading (standard coded 0 as reference), dummy coded race/ethnicity (Hispanic coded 0 as reference), grand mean centered body mass index [BMI] (x̄ = 29.27 kg/m^2^), dummy coded Meal-time variable (meal 1 as reference), dummy coded sex (male coded 0 as reference), insulin sensitivity of 5.15 mg/kg•min, and sleepiness, (x̄ = 35.12 mm).

* *p* < .05, ** *p* < .01, *** *p* < .001.

**Table 6:** Model assessing the insulin sensitivity moderation of the association of sleepiness with cravings for meat.^a^

Parameter	Main Effect	Within Person	Between Person	Interaction
Intercept	**3.07 (.35)** ^***^	**3.60 (.32)** ^***^	**3.60 (.32)** ^***^	**3.57 (.32)** ^***^
Age	.03 (.02)	.03 (.02)	.03 (.02)	.03 (.02)
Sex	**−1.17 (.36)** ^**^	**−1.20 (.35)** ^***^	**−1.20 (.35)** ^**^	**−1.12 (.35)** ^**^
Sequence	**−.89 (.33)** ^**^	**−.81 (.33)** ^*^	**−.81 (.33)** ^*^	**−.77 (.32)** ^*^
Race/Ethnicity	−.05 (.19)	−.11 (.19)	−.11 (.19)	−.09 (.19)
IS	.02 (.05)	.02 (.05)	.02 (.05)	.01 (.05)
Meal-time	**2.84 (.19)** ^***^	**2.88 (.19)** ^***^	**2.88 (.19)** ^***^	**2.87 (.19)** ^***^
Sleepiness	**.16 (.04)** ^***^	–	–	–
Meal-time^2^	**−.74 (.05)** ^***^	**−.74 (.05)** ^***^	**−.74 (.05)** ^***^	**−.74 (.05)** ^***^
W-P	–	**.11 (.04)** ^**^	–	**.11 (.04)** ^*^
B-P	–	–	**.38 (.09)** ^***^	**.36 (.09)** ^***^
W-P *IS	–	–	–	.01 (.01)
B-P*IS	–	–	–	**−.08 (.03)** ^*^
**Variance Components**				
Intercept (*u*_0i_)	3.04	2.87	2.87	2.72
Residual (*e*_ti_)	4.21	4.20	4.20	4.19

Abbreviations: IS = insulin sensitivity, W-P = within-person sleepiness, B-P = between-person sleepiness.

^a^ Values reported as unstandardized estimates (β) ±SE; the β values reflect rescaled scores. Intercept is interpretable at grand mean centered age (x̄ = 39 years), dummy coded caloric loading (standard coded 0 as reference), dummy coded race/ethnicity (Hispanic coded 0 as reference), grand mean centered body mass index [BMI] (x̄ = 29.27 kg/m^2^), dummy coded Meal-time variable (meal 1 as reference), dummy coded sex (male coded 0 as reference), insulin sensitivity of 5.15 mg/kg•min, and sleepiness, (x̄ = 35.12 mm).

* *p* < .05, ** *p* < .01, *** *p* < .001.

**Table 7:** Model assessing the insulin sensitivity moderation of the association of sleepiness with cravings for fruit.^a^

Parameter	Main Effect	Within Person	Between Person	Interaction
Intercept	**4.50 (.37)** ^***^	**5.24 (.34)** ^***^	**5.24 (.34)** ^***^	**5.19 (.34)** ^***^
Age	.02 (.02)	.02 (.02)	.02 (.02)	.02 (.02)
Sex	**−1.10 (.40)** ^**^	**−1.15 (.38)** ^**^	**−1.15 (.38)** ^**^	**−1.06 (.38)** ^**^
Race/Ethnicity	−.04 (.21)	−.14 (.21)	−.14 (.21)	−.11 (.20)
Sequence	−.69 (.36)	−.58 (.35)	−.58 (.35)	−.53 (.34)
IS	.10 (.06)	.09 (.06)	.09 (.06)	.09 (.05)
Meal-time	**1.12 (.18)** ^***^	**1.16 (.18)** ^***^	**1.16 (.18)** ^***^	**1.17 (.18)** ^***^
Sleepiness	**.22 (.04)** ^***^	–	–	–
Meal-time^2^	**−.31 (.04)** ^***^	**−.32 (.04)** ^***^	**−.32 (.04)** ^***^	**−.32 (.04)** ^***^
W-P	–	**.17 (.04)** ^***^	–	**.17 (.04)** ^***^
B-P	–	–	**.54 (.10)** ^***^	**.51 (.10)** ^***^
W-P *IS	–	–	–	−.01 (.01)
B-P*IS	–	–	–	**−.08 (.04)** ^*^
**Variance Components**				
Intercept (*u*_0i_)	3.87	3.54	3.54	3.38
Residual (*e*_ti_)	3.79	3.78	3.78	3.78

Abbreviations: IS = insulin sensitivity, W-P = within-person sleepiness, B-P = between-person sleepiness.

^a^ Values reported as unstandardized estimates (β) ±SE; the β values reflect rescaled scores. Intercept is interpretable at grand mean centered age (x̄ = 39 years), dummy coded caloric loading (standard coded 0 as reference), dummy coded race/ethnicity (Hispanic coded 0 as reference), grand mean centered body mass index [BMI] (x̄ = 29.27 kg/m^2^), dummy coded Meal-time variable (meal 1 as reference), dummy coded sex (male coded 0 as reference), insulin sensitivity of 5.15 mg/kg•min, and sleepiness, (x̄ = 35.12 mm).

* *p* < .05, ** *p* < .01, *** *p* < .001.

**Table 8:** Model assessing the insulin sensitivity moderation of the association of sleepiness with cravings for dairy food.^a^

Parameter	Main Effect	Within Person	Between Person	Interaction
Intercept	**4.30 (.37)** ^***^	**4.96 (.35)** ^***^	**4.96 (.35)** ^***^	**4.91 (.34)** ^***^
Age	−.01 (.02)	−.01 (.02)	−.01 (.02)	−.01 (.02)
Sex	**−1.02 (.40)** ^*^	**−1.07 (.39)** ^**^	**−1.07 (.39)** ^**^	**−.98 (.39)** ^*^
Race/Ethnicity	−.40 (.22)	**−.48 (.21)** ^*^	**−.48 (.21)** ^*^	**−.46 (.21)** ^*^
Sequence	**−.92 (.37)** ^*^	**−.82 (.36)** ^*^	**−.82 (.36)** ^*^	**−.77 (.35)** ^*^
IS	.11 (.06)	.10 (.06)	.10 (.06)	.10 (.06)
Meal-time	**.53 (.17)** ^**^	**.57 (.17)** ^**^	**.57 (.17)** ^**^	**.57 (.17)** ^**^
Sleepiness	**.19 (.04)** ^***^	–	–	–
Meal-time^2^	**−.16 (.04)** ^***^	**−.17 (.04)** ^***^	**−.17 (.04)** ^***^	**−.17 (.04)** ^***^
W-P	–	**.15 (.04)** ^***^	–	**.16 (.04)** ^***^
B-P	–	–	**.48 (.10)** ^***^	**.46 (.10)** ^***^
W-P *IS	–	–	–	−.01 (.01)
B-P*IS	–	–	–	**−.09 (.04)** ^*^
**Variance Components**				
Intercept (*u*_0i_)	4.13	3.86	3.86	3.67
Residual (*e*_ti_)	3.40	3.39	3.39	3.39

Abbreviations: IS = insulin sensitivity, W-P = within-person sleepiness, B-P = between-person sleepiness.

^a^ Values reported as unstandardized estimates (β) ±SE; the β values reflect rescaled scores. Intercept is interpretable at grand mean centered age (x̄ = 39 years), dummy coded caloric loading (standard coded 0 as reference), dummy coded race/ethnicity (Hispanic coded 0 as reference), grand mean centered body mass index [BMI] (x̄ = 29.27 kg/m^2^), dummy coded Meal-time variable (meal 1 as reference), dummy coded sex (male coded 0 as reference), insulin sensitivity of 5.15 mg/kg•min, and sleepiness, (x̄ = 35.12 mm).

* *p* < .05, ** *p* < .01, *** *p* < .001.

**Fig 2:**
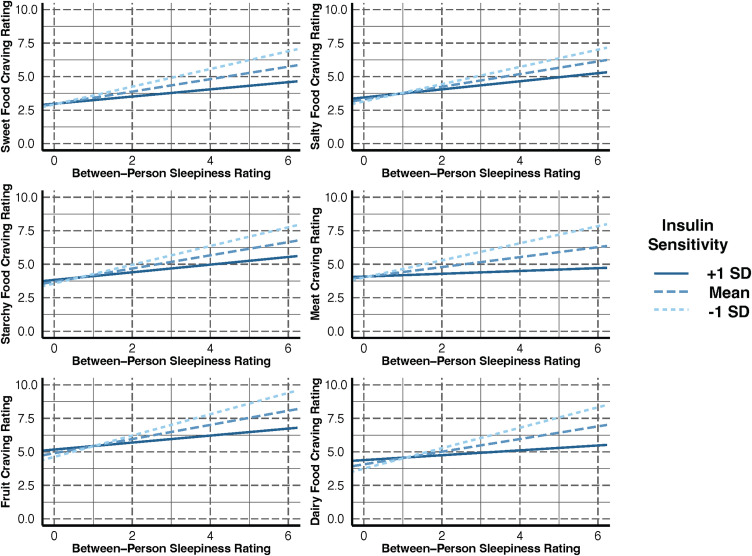
Simple slope analyses of between-person sleepiness ratings for each food craving. Fig 2 caption: Analyses supported the positive relationship of between-person sleepiness with all food cravings, except salty foods, was strengthened with more diminished insulin sensitivity. Specifically, for starchy foods, meat, fruit, and dairy foods the relationships were strengthened when insulin sensitivity was below-average, and average. For sweets, the association was strengthened when insulin sensitivity was below-average, average and above-average.

In contrast, the analyses of the moderation by insulin sensitivity of the between-person effect of sleepiness ratings on food cravings yielded the following significant interactions: sweet foods (β = ‑0.06, *p* < .05), starchy foods (β = ‑0.07, *p* < .04), meat (β = ‑0.08, *p* < .02), fruits (β = −0.09, *p* < .03), and dairy foods (β = ‑0.09, *p* < .02). The interaction for salty food cravings approached significance (β = ‑0.05, *p* < .09). Simple slope analyses of the significant interactions, as can be seen in Fig 2, showed that the positive relationship of between-person sleepiness with certain cravings for starchy foods, meat, fruit, and dairy foods was strengthened when insulin sensitivity was at below-average and average levels (starchy foods (β*(SE)*=0.69(.13), *p* < .001; β*(SE)*=0.47(.08), *p* < . 001), meat (β*(SE)*=0.64(.13), *p* < .001; β*(SE)*=0.37(.09), *p* < .001), fruit (β*(SE)*=0.80(.14), *p* < .001; β*(SE)*=0.52 (.10), *p* < .001), and dairy (β*(SE)*=0.74(.15), *p* < .001; β*(SE)*=0.44(.10), *p* < .001). For sweets, the relationship was strengthened when insulin sensitivity was at below-average, average, and above-average levels (β*(SE)*=0.66(.12), *p* < .001; β*(SE)*=0.46(.08), *p* < .001; β*(SE)*=0.26(.12), *p* < .05). Note, for salty foods, insulin sensitivity did not significantly moderate the association.

## Discussion

Sleep disorders, such as insomnia, short or insufficient sleep duration, and excessive daytime sleepiness have been identified as factors that may alter food preferences, and ultimately influence risk of obesity, prediabetes and type 2 diabetes [5,20–23]. The present study of non‑diabetic men and women utilized controlled experimental conditions, in circumstances resembling free-living, to confirm the positive association of daytime sleepiness with specific food cravings, and examined whether prevailing insulin sensitivity may interactively affect these associations. In sum, the primary study findings were that: **1)** prior to meal 1, fasting-level differences in cravings were observed with highest ratings for fruit, dairy foods, starchy foods, and meat, and with lowest ratings for salty and sweet foods; **2)** the change in cravings across meals over the day exhibited a quadratic trend for all food types, peaking at mid-day and then returning to pre‑meal 1 levels by the day’s end; **3)** the overall magnitude of craving ratings relative to zero did not differ between IS and IR groups, but the IS group displayed higher craving ratings relative to pre‑meal 1 level; **4)** from mid‑afternoon (i.e., meal 3) until the evening (i.e., meals 4 & 5), the IR participants reported less sleepiness than IS participants; and **5)** the positive association of sleepiness with most food craving ratings was accentuated in persons with average and below-average insulin sensitivity. Of note, these findings were independent of age, sex, race/ethnicity and meal administration sequence, which were controlled in analyses.

### Variation in food cravings

Highly palatable foods, rich in sugars, fats and salts, have high appeal and provide more rapid satisfaction than other food types [24,25]. Study participants expressed a desire for specific food types including fruit, dairy, starchy foods, and meat, more so than salty and sweet foods. It is notable, that these preferences were observed following an overnight fast, prior to the first meal, whereas the change of food cravings across the day did not differ among food types. Thus, the selective food preferences at meal 1 may reflect a desire not only for highly palatable foods, but also for foods that provide more calories. Studies have shown that visual and olfactory food-cue exposure increases physiological responses and food cravings, as well as food consumption [26]. In the present study, food-craving ratings were performed in the absence of external food-related cues. Therefore, it is not likely that such cues were responsible for the food-craving differences observed. However, craving ratings may have been influenced by interoceptive cues, such as hormonal signals, metabolic factors, and motivation-reward pathways linked with food-related cognitions [27].

The observed quadratic food craving pattern across the day is consistent with a well‑established literature linking circadian rhythms, entrained by light synchronizing cues, with physical activity and food consumption [28]. Scheer and colleagues [29] used a controlled in‑laboratory 13-day design to examine specific food-type appetites, similar to that assessed in the present study. Participants reported lowest food-craving levels in the morning, and then an elevation in food cravings throughout the day, maximizing in the mid‑evening. The current data were comparable in that food cravings were lowest in the initial waking hours. This appetite suppression has been posited to result from an endogenous circadian influence, separate from sleep/wake cycling, and possibly influenced by hormonal factors, such as ghrelin, which is lower in the morning [30]. In contrast to the findings of Scheer and colleagues [29], present analyses showed that, from the mid-day peak, food cravings declined in the afternoon and evening hours. It is likely that, in our sedentary laboratory environment, caloric intake exceeded physical requirements, and by the afternoon, food cravings were blunted [31].

### Insulin sensitivity and the association of sleepiness with food cravings

It might be expected that, in the context of insulin resistance, with higher blood glucose and insulin levels, hunger may be higher. However, compared with IR counterparts, the IS group reported 36% greater food-craving ratings over the day relative to meal 1 level. We previously documented that the IR group displayed virtually all of the anthropometric and metabolic features that cluster with insulin resistance [15]. Analyses from this study showed that the IR participants displayed a compensated insulin hypersecretion and enhanced beta-cell glucose sensitivity associated with diminished insulin-mediated glucose uptake from the early to the late postprandial phase. It is probable that hormonal signaling (e.g., ghrelin, leptin, GLP-1) played a role in differentially lowering the hunger and specific appetites of the IR group [32,33]. The present results would suggest that such signaling may also reflect a compensatory mechanism to reduce hunger in persons with preclinical insulin resistance. Of note, insulin transport into the cerebrospinal fluid is attenuated in persons with diminished insulin sensitivity [34]. In addition, a previous CNS-imaging study of non-diabetic individuals showed that intranasal insulin infusion, as mediated by mesolimbic pathways, induced lower food preference ratings, and that this effect was strengthened with lower insulin sensitivity [35]. Moreover, others have found that insulin resistance is associated with an alteration of hypothalamic insulin and leptin post-receptor mechanisms [36–39]. Thus, it may be posited that the valuation of food stimuli and, consequently, food seeking and consumption, may be altered by central biomechanisms in the context of insulin resistance. Further research is needed to examine hormonal signaling and avenues of central insulin transport as potential mechanisms underlying the observed relationships.

Insulin resistance has been associated with insufficient sleep, habitual short sleep, and other sleep conditions, such as insomnia [40,41]. Thus, it is notable that, compared with the IS group, the IR participants reported, not higher, but lower sleepiness later in the day than the IS group. To our knowledge this is a novel finding. Many reports linking insulin resistance with sleep dysfunction have been studies of type 2 diabetic cohorts, wherein the chronicity and severity of disease, and related sequelae, may deleteriously influence sleep function. The IR individuals of the present study, like IS counterparts, had not been previously diagnosed with diabetes or other confounding conditions. Thus, the present finding suggests that preclinical insulin resistance may be associated with an inhibition or delay in the afternoon increase in sleepiness that has been previously documented in persons without diabetes [13]. Further study is necessary to eliminate measurement error or variation in other biomechanisms including circadian function as possible explanations for this finding.

The prior literature highlighting the relationship between sleep function with food preferences suggested that this association might be more limited to specific food types [8,41]. However, study results showed that with increased sleepiness, increased desire for all food types was observed. This finding appears to contradict the hypothesis that daytime sleepiness restrictively stimulates cravings for sweets and more calorie dense foods [42]. Of note, even though the IR participants reported less sleepiness later in the day than IS participants, and lower food cravings than IS participants, the present study shows that elevated daytime sleepiness may exert a greater effect on the desire for food in persons with average and below-average insulin sensitivity. Additional research is needed to delineate underlying mechanisms supporting the linkage of sleep function and food cravings with food consumption and metabolic dysregulation early in diabetes pathophysiology.

### Limitations and strengths

The study, which enrolled a nonclinical sample, was strengthened by applying strict eligibility criteria, and by statistically controlling for key potential confounding variables. These procedures reduced the possibility that findings were confounded by structural and functional effects of sleep dysfunction, chronic disease, or medical treatment. The strict eligibility criteria, thus, biased against detecting significant relationships in the present study. Yet, theoretically reasonable findings were observed. Study findings should be considered independent of age, sex, race/ethnicity and meal administration sequence, which were controlled in analyses. However, the majority of the study sample included Hispanic/Latino participants. Thus, the generalizability of the current findings to other race/ethnicity groups is not clear because it is possible that sociocultural factors unique to Hispanic/Latino men and women may have influenced observed dietary preferences [43]. In addition, it should be noted that body fat deposition, indexed by BMI, was not controlled in the analyses due to its collinearity with insulin sensitivity, which violates the assumption of independence required for its inclusion. BMI is often a surrogate marker for adiposity, and prior research has shown that insulin sensitivity is strongly influenced by body fat distribution, particularly visceral fat, which is a key determinant of metabolic health [44]. Further study is needed to delineate the extent to which adiposity or some related biomechanism, independent of insulin metabolic regulation, may have accounted for the present study findings.

Although no participants reported having a previously diagnosed sleep disorder, it is possible that habitual sleep function and circadian rhythm effects, even within normative ranges, influenced daytime sleepiness perception. Thus, further study of these factors is warranted to confirm the present findings. The order for surveying food type cravings was fixed to ensure adherence to validated instrument guidelines and preserve comparability with prior research. However, doing so, may have favored a conclusion that food-craving ratings were less selective and more a reflection of general hunger. In addition, the analytic strategy to use multiple separate models may have increased the Type I error. Therefore, in this respect, findings should be viewed with appropriate caution. Nevertheless, the study findings yielded systematic differences in the positive association of sleepiness with food cravings that were consistent with previous findings and with the putative role of insulin sensitivity in this association. Of note, study conclusions pertaining to food seeking and consumption were limited because food cravings were restricted to specific food types. A more complete examination of food-related behavior would involve an evaluation of food palatability, the response‑cost required to obtain the food, as well as the food amount consumed [44]. Thus, further study is necessary to determine whether the observed interaction of insulin sensitivity with the association of sleepiness with food preferences directly relate to engagement in food-related behaviors aligned with those preferences.

## Conclusion

In sum, this study provides preliminary evidence that insulin sensitivity moderates the sleepiness-craving relationship. Specifically, in this study of nondiabetic men and women, findings showed that the sleepiness-craving associations for a range of food types are stronger when insulin sensitivity is more diminished. Thus, findings suggest that, well before frank diabetes occurs, people may experience different food appetites not only by being sleepier than others, but also as a function of their metabolic regulatory status.

## Supporting information

S1 File Hypothesized model wherein sleepiness ratings predict specific food cravings, moderated by insulin sensitivity, as an integrated metabolic status marker that includes adiposity-related variance. **Note.** Abbreviations: IS = insulin sensitivity. Sequence is the order of caloric intake day, such that participants received either the ‘standard’ day followed by ‘high’ day, or the ‘high’ day followed by ‘standard’ day. Of relevance, body mass index (BMI) was not used as a covariate in the analyses because BMI is strongly confounded with insulin-resistance status, and, therefore, adjusting for BMI would constitute overadjustment and would remove variance that is part of the causal pathway from adiposity → insulin resistance → outcome. In the present data, BMI differed significantly between the insulin-resistant (IR) and insulin-sensitive (IS) groups (p < .001), indicating that BMI was not evenly distributed across the factor levels and was instead intrinsically related to group membership. In addition, BMI was strongly associated with insulin sensitivity measured by the gold-standard M-value as obtained using the euglycemic-hyperinsulinemia clamp. After controlling for age, sex, sequence, and race/ethnicity, BMI remained significantly correlated with M (partial r = −0.54, p < .001), demonstrating that BMI shared substantial variance with the physiological construct that defines the IR status grouping variable.
(DOCX)
