# Supplementary material for: Daytime sleepiness and specific food cravings: The moderating role of insulin sensitivity
Source: PLoS One. 2026 Mar 9;21(3):e0343407. doi: 10.1371/journal.pone.0343407 (PMC12970934; doi:10.1371/journal.pone.0343407)
Supplement: S1 File — Note. Abbreviations: IS = insulin sensitivity. Sequence is the order of caloric intake day, such that participants received either the ‘standard’ day followed by ‘high’ day, or the ‘high’ day followed by ‘standard’ day. Of relevance, body mass index (BMI) was not used as a covariate in the analyses because BMI is strongly confounded with insulin-resistance status, and, therefore, adjusting for BMI would constitute overadjustment and would remove variance that is part of the causal pathway from adiposity → insulin resistance → outcome. In the present data, BMI differed significantly between the insulin-resistant (IR) and insulin-sensitive (IS) groups (p < .001), indicating that BMI was not evenly distributed across the factor levels and was instead intrinsically related to group membership. In addition, BMI was strongly associated with insulin sensitivity measured by the gold-standard M-value as obtained using the euglycemic-hyperinsulinemia clamp. After controlling for age, sex, sequence, and race/ethnicity, BMI remained significantly correlated with M (partial r = −0.54, p < .001), demonstrating that BMI shared substantial variance with the physiological construct that defines the IR status grouping variable. (DOCX) [file pone.0343407.s001.docx]

**S1. Hypothesized model wherein sleepiness ratings predict specific food cravings, moderated by insulin sensitivity, as an integrated metabolic status marker that includes adiposity-related variance**
